# Supplementary material for: Effect of Agitation and Temporary Immersion on Growth and Synthesis of Antibacterial Phenolic Compounds in Genus Drosera
Source: Biomolecules. 2024 Sep 7;14(9):1132. doi: 10.3390/biom14091132 (PMC11430277; doi:10.3390/biom14091132)

Effect of Agitation and Temporary Immersion on Growth and Synthesis of Antibacterial Phenolic Compounds in Genus *Drosera*

Wojciech Makowski [1,*], Kinga Mrzygłód [1], Agnieszka Szopa [2], Paweł Kubica [2], Marta Krychowiak-Maśnicka [3], Krzysztof Michał Tokarz [1], Barbara Tokarz [1], Iga Ryngwelska [1], Ewa Paluszkiewicz [4] and Aleksandra Królicka [3,*]

1 Department of Botany, Physiology and Plant Protection, Faculty of Biotechnology and Horticulture,
University of Agriculture in Krakow, 29 Listopada 54, 31-425 Krakow, Poland;
salome269.40@gmail.com (K.M.); krzysztof.tokarz@urk.edu.pl (K.M.T.);
barbara.tokarz@urk.edu.pl (B.T.); iga.ryngwelska788@gmail.com (I.R.)

2 Department of Pharmaceutical Botany, Collegium Medicum, Jagiellonian University, Medyczna 9,
30-688 Krakow, Poland; a.szopa@uj.edu.pl (A.S.); p.kubica@uj.edu.pl (P.K.)

3 Laboratory of Biologically Active Compounds, Intercollegiate Faculty of Biotechnology UG and MUG,
University of Gdansk, Abrahama 58, 80-307 Gdansk, Poland;
marta.krychowiak@ug.edu.pl

4 Faculty of Chemistry, Gdansk University of Technology, Narutowicza 11/12, 80-233 Gdansk, Poland;
ewapalus@pg.edu.pl

* Correspondence: wojciech.makowski@urk.edu.pl (W.M.);
aleksandra.krolicka@ug.edu.pl (A.K.)

Supplementary Data S1.

^{1}H NMR Spectra for 8-chloro-5-hydroxy-7-methyl-1,4-naphthoquinone

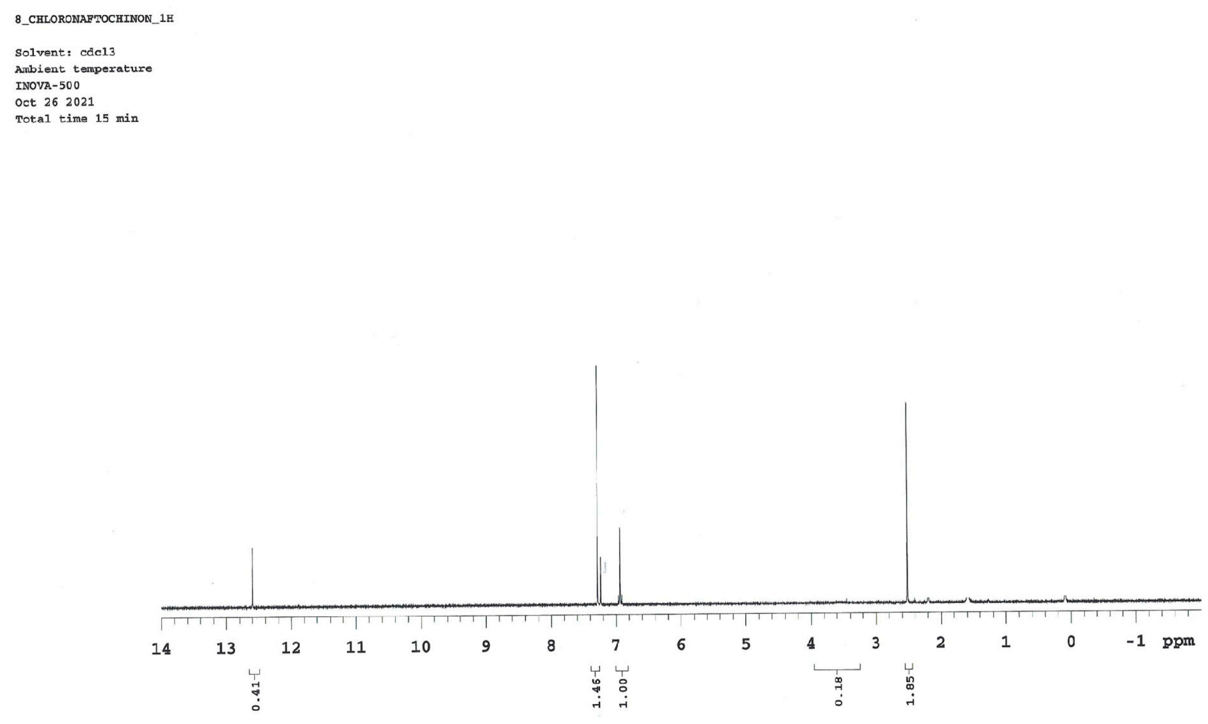

^{1}H NMR Spectra for 5-hydroxy-7methyl-1,4-naphthoquinone (Ramentaceone)

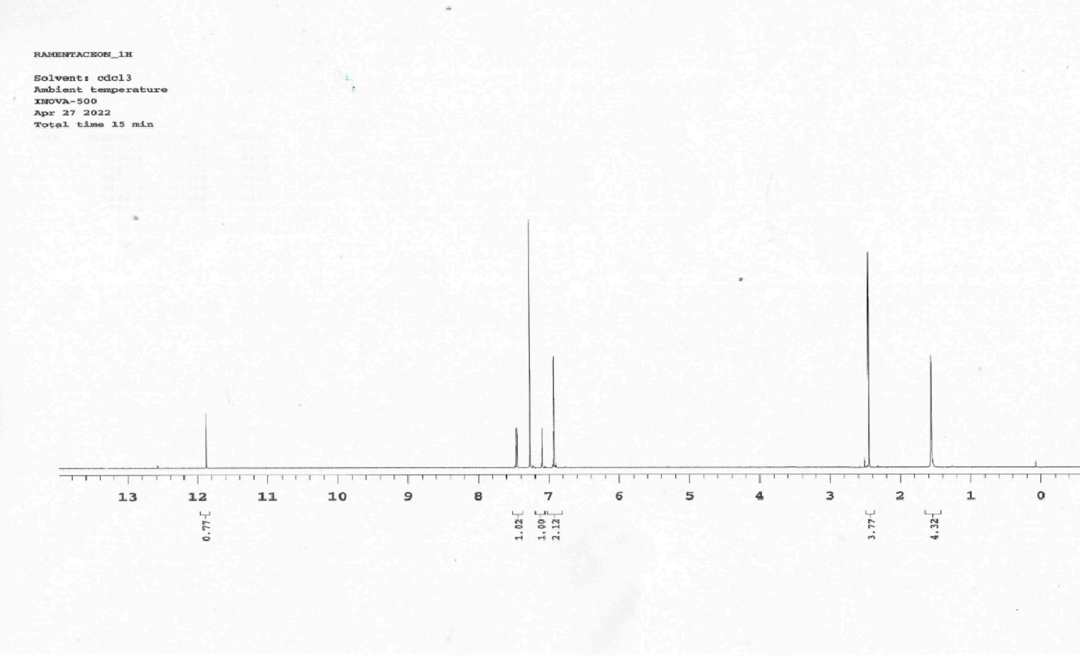

ESI-MS Spectra for 5-hydroxy-7methyl-1,4-naphthoquinone (Ramentaceone)

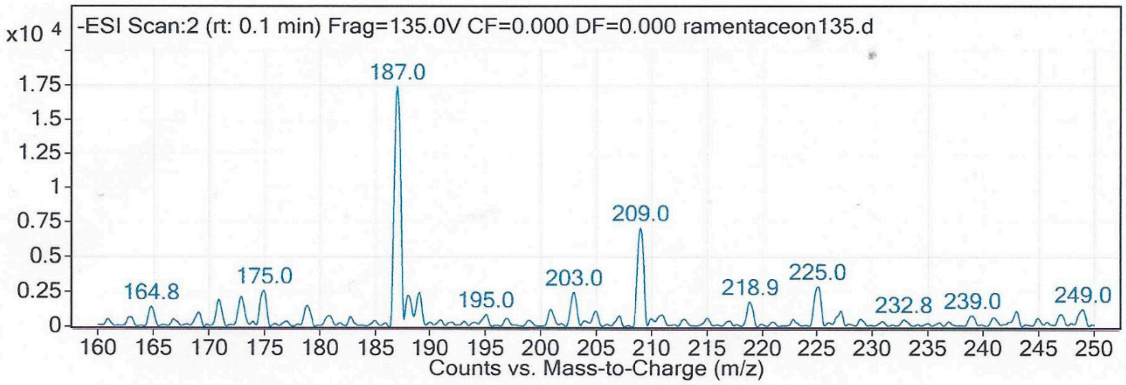

Supplement: Supplementary file 1 [file biomolecules-14-01132-s001.zip › biomolecules-3157381-supplementary.pdf]
